# Nipah Virus in the Fruit Bat *Pteropus vampyrus* in Sumatera, Indonesia

**DOI:** 10.1371/journal.pone.0069544

**Published:** 2013-07-22

**Authors:** Indrawati Sendow, Atik Ratnawati, Trevor Taylor, R. M. Abdul Adjid, Muharam Saepulloh, Jennifer Barr, Frank Wong, Peter Daniels, Hume Field

**Affiliations:** 1 Indonesian Research Center for Veterinary Science, Bogor, Indonesia; 2 Australian Animal Health Laboratory, Geelong, Victoria, Australia; 3 Queensland Centre for Emerging Infectious Diseases, Department of Agriculture, Fisheries and Forestry, Coopers Plains, Queensland, Australia; 4 EcoHealth Alliance, New York, New York, United States of America; University of Pretoria, South Africa

## Abstract

Nipah virus causes periodic livestock and human disease with high case fatality rate, and consequent major economic, social and psychological impacts. Fruit bats of the genus *Pteropus* are the natural reservoir. In this study, we used real time PCR to screen the saliva and urine of *P. vampyrus* from North Sumatera for Nipah virus genome. A conventional reverse transcriptase (RT-PCR) assay was used on provisionally positive samples to corroborate findings. This is the first report of Nipah virus detection in *P. vampyrus* in Sumatera, Indonesia.

## Introduction

Nipah virus (NiV) is a novel paramyxovirus that is pathogenic to pigs and humans. The first attributed outbreak was reported in 1998–9 in Malaysia, causing respiratory disease in pigs. Humans were subsequently infected, with a high case fatality rate. Fruit bats of the genus *Pteropus* (commonly known as ‘flying-foxes’) are the putative natural reservoir host [1], [2]. Comparative genomic analyses have identified NiV as a henipavirus, closely related to Hendra virus (HeV) [3].

Johara *et al,* 2001 [1] suggested (based on their serologic findings) that flying-foxes were the likely natural reservoir of Nipah virus in Malaysia, and beyond. This contention was strengthened following the detection of Nipah virus genome in the urine and saliva of *P*. *hypomelanus* and *P. vampyrus* in Malaysia [2], [4]. Nipah infection has subsequently been reported in *Pteropus spp.* across their global distribution, strongly suggesting that Nipah and related viruses have a long association with bats of this genus [5].

Since the outbreak in Malaysia in 1998, Nipah virus has been reported in Bangladesh and India. Human fatalities have occurred in Bangladesh nearly every year since 2001 [6]–[8], and in India in 2001 and 2007 [9].

Henipaviruses have a broad mammalian host range, thus in Asia at least, Nipah is regarded as a potential zoonotic disease requiring strategic preparedness and response. Anti-Nipah virus antibodies have now been identified in *Pteropus* or other bat species in Cambodia [10], Thailand [11], Indonesia [12], India [13], China [14], Vietnam [15], Bangladesh [6], Madagasgar [16] and Ghana [17]; viral genome has been detected in bats in Malaysia (*P. hypomenalus, P. vampyrus*) [4], [18], Cambodia (*P. lylei*) [19], Thailand (*P. lylei*, *P. hypomelanus*, *P. vampyrus*) [11], [20], [21], India (*P. giganteus*) [22] and Ghana (*Eidolon helvum*) [23]. In this paper we report the detection of Nipah virus genome in *P. vampyrus* in Sumatera, Indonesia using real time PCR.

## Methods

### Ethics Statement

All animal work was conducted according to relevant national guidelines. At the time (2009), it was not a requirement to obtain animal ethics approval to collect samples from captive flying-foxes in Indonesia. All animals were handled humanely.

### Sample Collection

Samples were sourced from flying-foxes (*P. vampyrus*) for sale in animal markets in two locations (Kota Medan (KM) and Deli Serdang (DS)) in northern Sumatera between 25 and 29 May, 2009. Individual flying-foxes were physically restrained, and saliva samples collected by oro-pharyngeal swab. Pooled urine (PU) samples were collected by placing plastic sheeting under one or more cages each containing multiple (3–8) flying-foxes. The size of the cages varied from 1–1.5 m×1.5–2 m (wholesaler) to 40–70 cm×50–70 cm (market seller). We typically had multiple cages over each sheet, with each cage containing five to eight bats. The urine was subsequently syringed from the sheet and placed in sterile tubes. In addition, the urinary bladder (UB) was collected from flying-foxes butchered in the market, and stored in Dubelco’s Minimun Essential Media (DMEM) with antibiotic Kanamycin and 2% Foetal Bovine Serum (FBS). A 2 ml blood sample was also collected post-mortem by cardiac puncture.

### Nucleic Acid Extraction

Briefly, RNA was extracted from 100 µl samples of pooled urine, 10% tissue homogenates, bladder urine, oro-pharangeal swabs and urogenital swabs using an RNeasy kit (QIAGEN) according to the manufacturer’s protocols. RNA was diluted in 50 ul of RNase free H_2_O and stored at −80°C prior to use.

### Primers and Probe for TaqMan Assay

The TaqMan PCR assay for the detection of Nipah virus N gene used primers and a probe designed at the Australian Animal Health Laboratory, Geelong, Australia (AAHL) (Pritchard personal communication). The assay used the primers (Nipah-N1198F (5′-TCAGCAGGAAGGCAAGAGAGTAA-3′), Nipah-N1297R (5′-CCCCTTCATCGATATCTTGATCA-3′)) and the 5-carboxyflourescein (FAM) labeled probe (Nipah-1247comp-FAM (5′–CCTCCAATGAGCACACCTCCTGCAG-3′)) specific for Nipah virus. TaqMan ribosomal RNA control reagents (Applied Biosystems, Foster City, CA) were incorporated to validate the RNA extraction procedure, determine the integrity of the RNA sample and the absence of significant level PCR inhibitors. The control contained 6-caboxyrhodamine (VIC)-labelled probe specific for any eukaryotic 18S rRNA and was performed as a primer-limited multiplex reaction in each sample.

### TaqMan RT-PCR Assays

One-step RT-PCR reactions were performed using the TaqMan one-step RT-PCR kit (applied Biosystems) in a 25 µl total reaction mix. Each sample was tested in triplicate. 2 µl of viral RNA was added to 23 µl of the reaction mix. The assay was performed in an ABI 7300 PCR thermocycler with the following parameters 30 min at 48°C, 10 min at 95°C, and 45 cycles of 15 sec at 95°C and 1 min at 60°C. A positive result in the Real Time PCR is indicated by a characteristic amplification plot with a threshold set at 0.05 to 0.1 and a CT value of under 37. Results with a CT value between 37 and 40 were considered to be indeterminate and were retested. A CT value of 40 and above was considered a negative result.

### Reverse Transcriptase PCR

Reverse transcriptase PCR targeting the M gene was performed using Superscript III One-Step RT-PCR with Platinum Taq (Invitrogen) and specific primers Nipah Primer 2 (5 ′TGGAATCTACATGATTCCAAGAACCATG 3′) and Nipah 3C (5 ′TAATGTGGAGACTTAGTCCGCCTATG 3′). 10 µl of DNA was added to 40 µl of Master Mix. The PCR was performed in an ABI 9700 PCR thermocycler with reaction conditions: 1 min at 94°C, 2 min at 37°C, and 40 cycles of 2 minutes at 72°C and 15 minutes at a temperature of 70°C. The products produced by RT-PCR were visualized by performing electrophoresis using a 1.5% agarose gel. Amplification using these primers produced a PCR product of 279 bp. Extracts yielding a positive result on either PCR assay were forwarded to the CSIRO Australian Animal Health Laboratory (AAHL) for corroboration.

### Sequencing

PCR products obtained at the Indonesian Research Centre for Veterinary Science (IRCVS) were sent to AAHL for further analysis. At AAHL, the PCR products were amplified using primers that were internal to Nipah primer 2 and Nipah primer 3C. The primers NipahTT1 and NipahTT2 had the sequences NipahTT1 CGCCTATGGAACCCAGTG and NipahTT2 TCCACGAACCATGCTTGA. The resulting PCR products were then gene cleaned and sequenced. Sequencing was performed in an ABI 3130XL Genetic Analyzer using a BigDyeTerminator V3.1 cycle sequencing kit. Each sequence determined was analyzed and aligned using the SeqMan Pro module of the Lasergene ver. 8.0.2 software package. The sequence was used as a query in a BLAST 2.2.23 search to obtain identification.

### Serology

Serology used the Multiplex Microsphere Binding assay as described by Bossart *et al,* 2007 [24]. Briefly, carboxylated microspheres (Luminex corp.) were covalently coupled to soluble recombinant G protein of both HeV and NiV. The beads were blocked using 100 µl 2% skim milk/PBS-T and shaken for 30 min at room temperature. After vacuum removal of the liquid, 100 µl of test sera diluted 1∶50 in PBS-T was mixed with the beads and shaken for 30 min at room temperature. After vacuum removal of the liquid, 100 µl of biotinylated Protein A/G (Pierce) diluted 1∶500 in PBS-T was mixed with the beads and shaken for 30 min at room temperature. After vacuum removal of the liquid, 100 µl streptavidin phycoerythrin (Qiagen) diluted 1∶1000 in PBS-T was mixed with the beads and again shaken for 30 min at room temperature. The Median Fluorescent Intensity (MFI) values were read by the BioRad BioPlex machine. Samples with an MFI above 200 were considered potentially positive, and samples with an MFI above 1000 were considered strongly positive [24].

## Results

### Samples

**Figure 1:**
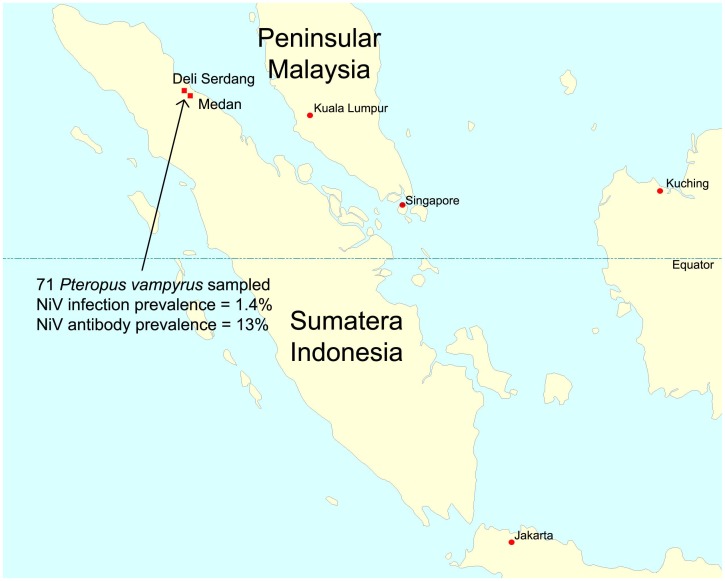
Map of Indonesia showing the island of Sumatera and indicating the sampling locations.

A total of 215 samples (71 oro-pharangeal swabs, 71 blood samples, 32 pooled urine samples and 41 urinary bladder samples) were collected from 71 *P. vampyrus* flying-foxes from two locations (Kota Medan and Deli Serdang Kampung) in the Indonesian province of North Sumatera (Figure 1) (Tables 1 and 2).

**Table 1:** Realtime PCR results in *P. vampyrus* samples from North Sumatera, 2009^1^.

Location	Sample type	Number	Number (%) PCR positive
Kota Medan	Pooled urine	10	0 (0%)
	Urinary bladder	14	0 (0%)
	Oro-pharangyeal swab	24	0 (0%)
Deli Serdang	Pooled urine	22	2 (9%)
	Urinary bladder	27	1 (4%)
	Oro-pharangyeal swab	47	1 (2%)
Total pooled urine		32	2 (6%)
Total oro-pharangeal swab		71	1 (1.5%)
Total urinary bladder		41	1 (2.5%)

1 A fifth sample (pooled urine sample PU21) yielded a ‘trace’ result by real-time PCR but was negative by conventional PCR, and is not included here.

**Table 2:** Luminex serology on seventy-one^1^ sera collected from Kota Medan and Deli Serdang.

Kota Medan			Deli Serdang		
**1**	97.5	892	**1**	152.5	984
**2**	73.5	79	**2**	96.5	812.5
**3**	95	1162	**3**	84	89
**4**	83	793.5	**5**	89	95
**5**	78	206.5	**6**	80	79
**6**	93	110.5	**7**	200	5862
**7**	81	96	**8**	87.5	86.5
**8**	387	540	**9**	89	365
**9**	80	154	**11**	61	63
**10**	78	73	**12**	70	385
**11**	73	71	**13**	72.5	83
**12**	95.5	80	**14**	82.5	804.5
**13**	82	83	**15**	84.5	102
**14**	97	91	**16**	89	97
**15**	73	76	**17**	63	186
**16**	76	139	**18**	498	2847
**17**	73.5	183.5	**19**	122	537
**18**	78.5	81	**20**	285	437
**19**	413.5	5701.5	**21**	86	578
**20**	173.5	3837	**22**	329	1665.5
**21**	74	137	**23**	370.5	5421
**22**	80	666	**24**	90.5	98
**23**	78	85.5	**25**	81	553
**24**	78	79	**26**	71	83
			**27**	80	133
			**28**	69	84
			**29**	85	73
			**30**	121.5	1605
			**31**	77.5	551.5
			**32**	79	94
			**33**	89.5	67
			**34**	56	142.5
			**35**	92	1244.5
			**36**	79.5	89
			**37**	70	142.5
			**38**	82	303.5
			**39**	85.5	570
			**40**	59.5	90.5
			**41**	78.5	86
			**42**	77.5	874
			**43**	75	757
			**44**	76	90.5
			**45**	71	97
			**46**	60	164
			**47**	65.5	247.5

Sera with an MFI over 1000 were defined as strongly positive (denoted with a solid underline). Sera with an MFI over 200 were defined as potentially positive (denoted with a dashed underline).

1 Two samples were unsuitable for testing.

### PCR Detections

**Figure 2:**
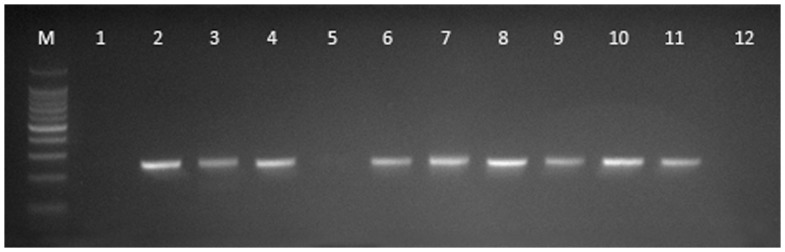
Conventional RT-PCR gel (M = 100 bp markers, 1 = UB21 undiluted, 2 = UB21 1∶10 dilution, 3 = PU21 undiluted, 4 = PU21 1∶10 dilution, 5 = DS21 undiluted, 6 = DS21 1∶10 dilution, 7 = PU18 undiluted, 8 = PU18 1∶10 dilution, 9 = PU20 undiluted, 10 = PU20 1∶10 dilution, 11 = Positive control, 12 = No template control).

Four samples yielded Nipah virus genome by real-time (Table 1) and conventional PCR (Figure 2): an oro-pharangeal swab and a bladder sample from DS 21, and two pooled urine samples (PU 18 and PU 20). The positive pooled urine samples included urine from bats DS 1 to DS 22. The findings were externally corroborated by the CSIRO Australian Animal Health Laboratory. A fifth sample (pooled urine sample PU21) yielded a ‘trace’ result by real-time PCR but was negative by conventional PCR.

### Sequencing and Sequence Analyses

**Figure 3:**
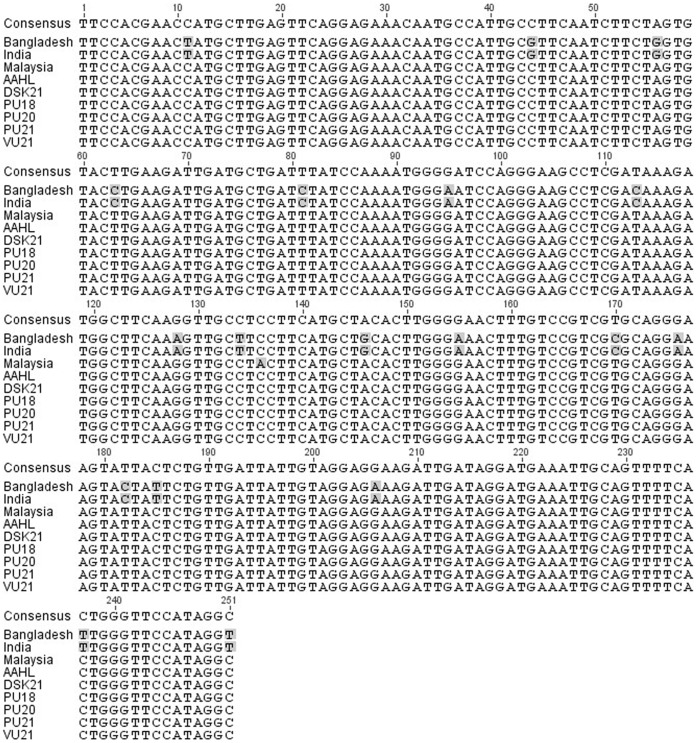
Alignment of a 251 nt sequence region of the Nipah virus matrix (M) gene from our Indonesian bat (P. vampyrus/2013/Genbank KC903168-KC903172), the AAHL reference virus (Malaysia/human/1999/Genbank AF212302), a Malaysian bat (P. vampyrus/2010/Genbank FN869553), Bangladesh (human/2004/Genbank AY988601) and India (human/2007/Genbank FJ513078).

Sequencing at AAHL confirmed the products as Nipah virus. Analyses showed that our nucleotide sequence had 100% alignment with the AAHL reference virus (Malaysia/human/1999/Genbank AF212302), 99.6% homology with the Malaysian bat sequence (P. vampyrus/2010/Genbank FN869553), and 92.8% homology with the Bangladesh (human/2004/Genbank AY988601) and India (human/2007/Genbank FJ513078) sequences over the region sequenced (Figure 3). Amino acid sequences were compared for the Indonesian and Malaysian bats, with the 99.6% nt homology translating to one amino acid substitution over the sequenced region.

### Serology

A total of seventy-one serum samples were collected from the two locations. Two samples were unsuitable for testing. Twenty-nine sera (42%) had an MFI of 200 or greater in the NiV assay (Table 2). Of these, nine (13%) had an MFI over 1000, indicating strong reactivity to NiV sG. Seven of these had low cross-reactivity to HeV sG.

## Discussion

In this study, we targeted saliva and urine samples from *Pteropus vampyrus* in northern Sumatera based on the findings of Johara *et al,* 2001 [1] and Chua *et al,* 2002 [18], and adapting the methods of Wacharapluesadee *et al,* 2005, 2006 [11], [20]. Four samples yielded Nipah virus genome - an oro-pharangeal swab and a bladder sample from DS 21 (an adult female), and two pooled urine samples containing urine from DS 21. The positive individual samples from DS 21 clearly indicate she was excreting virus, and with her urine contributing to the positive pooled urine samples, it is probable that DS 21 was the only one individual excreting virus at the time of sampling, translating to an infection/excretion prevalence of 1.4% (95% CI 0.04–7.6%). This prevalence estimate should not be over-interpreted – our study was a cross-sectional ‘point in time’ survey, and prevalence will likely vary with a range of factors over time, as indicated by the 95% confidence interval. Previous studies have suggested a positive association between pregnancy and henipavirus infection history, and while this individual was a sexually mature female, her pregnancy status was unknown.

Collecting urine from individual flying-foxes can be difficult and urine volume can be limited. Using plastic sheeting under roosting bats to facilitate the collection of pooled urine samples is an effective and efficient way to collect urine samples, and an effective way to detect henipaviruses [25], [26]. We contend that the potential for failed detection of positive individual urines as a result of dilution with negative individual urines is negated or minimized by the sensitivity of current PCR techniques. Further, at a population level, collecting pooled urine samples under roosting flying-foxes means that a greater number of individuals are being sampled, increasing the likelihood of detection when infection prevalence is low.

Our study further supports Nipah virus excretion in urine, consistent with the findings of Wacharapluesadee *et al*, 2005 [11] and Rahman *et al*, 2010 [4], in free-living naturally infected flying-foxes, and Middleton *et al*, 2007 [27] in experimentally infected captive flying-foxes. The findings also underline the value of the pooled urine sampling methodology as a means of detecting and characterizing bat henipaviruses. The detection in urinary bladder (vs urine) is novel (though not unexpected), and may offer a diagnostic option when a urine sample is not present at necropsy, or when the sampling strategy targets wet markets.

Virus isolation was not undertaken. Nipah virus is categorized as a BSL 4 agent, and Indonesia does not currently have a laboratory with BSL4 facilities. Realtime PCR and RT- PCR represent a practical and robust alternative to detect Nipah virus from field samples in this situation [21]. The assays target the N and M genes respectively, both of which are highly conserved among henipaviruses [28], allowing confident identification of Nipah virus from field samples rapidly and specifically [29].

Our analyses showed that the Indonesian and Malaysian nucleotide sequences were more closely aligned that sequences with each other than they were with the Bangladesh or Indian sequences. This is not unexpected given the demonstrated movement of flying-foxes between peninsular Malaysia and Sumatera (Figure 1) across a sea distance of less than 50 km [30]. While it might be argued that the weaker alignment with the Bangladesh and Indian sequences reflects the non-flying-fox origin of the latter, analysis of sequence derived from multiple species in Malaysia suggests distinct geographic clades [4]. Sequence comparison across a larger portion of the genome, and from a broader geographic footprint across Indonesia is needed to determine the extent of genetic diversity in Indonesian flying-foxes, especially East Indonesia.

The serology findings corroborate those of Johara *et al*, 2001 [1], Sendow *et al,* 2006 [12] and Rahman *et al* 2013 [31] and indicate that Nipah virus and potentially cross-reacting henipaviruses are endemic in *P. vampyrus* across their geographic range.

### Conclusion

Nipah virus generates considerable concern in Asia, both in relation to veterinary health and public health. While no incidents in livestock or humans has been recorded since those in Malaysia and Singapore in 1998–99, the associated economic and social impacts are well remembered in the region, periodically refreshed by incidents in Bangladesh. Previous studies have demonstrated anti-Nipah virus antibodies in flying-foxes in Indonesia; this study provides the first molecular evidence that Nipah virus indeed circulates in populations of flying-foxes in Indonesia. Further, we show that the virus is indistinguishable from that detected in *P. vampyrus* in peninsular Malaysia, which (notwithstanding the desirability of additional genomic sequence) supports the likelihood that there is a single regional mega-population of *P. vampyrus*, and that flying-foxes move unconstrained across national boundaries. These findings can hopefully inform regional policy and strengthen emerging diseases awareness and preparedness in Indonesia and region.
